# Scleral Lens Fenestrations and Fluid Reservoir Debris

**DOI:** 10.1007/s44402-026-00150-z

**Published:** 2026-07-25

**Authors:** Damien Fisher, Luisa Holguin Colorado, David Alonso-Caneiro, Stephen J. Vincent

**Affiliations:** 1 Centre for Vision and Eye Research, Optometry and Vision Science, Queensland University of Technology, Brisbane, Queensland Australia; 2 School of Science, Technology and Engineering, University of the Sunshine Coast, Petrie, Queensland Australia

**Keywords:** Fenestration, Fluid reservoir debris, Midday fogging, Scleral lens

## Abstract

**Purpose:**

The accumulation of debris within the fluid reservoir during scleral lens wear can adversely affect vision, resulting in the need for lens removal. This study aimed to quantify the effect of incorporating limbal scleral lens fenestrations upon fluid reservoir debris.

**Methods:**

Twenty healthy adults (mean age 32 ± 11 years, 10 females/10 males) with normal corneas wore non-fenestrated and fenestrated (3 × 1 mm diameter limbal fenestrations separated by 120°) OneFitMed+ scleral lenses (No.7 Contact Lenses) in both eyes (on separate days) for 3 h. All other scleral lens parameters were held constant except for the addition of the fenestrations by the manufacturer. Customised image analysis (MATLAB) was used to quantify central fluid reservoir debris (an average and total intensity metric) from Scheimpflug images of the right eye.

**Results:**

Fluid reservoir debris increased over time (*p* < 0.001), more so for non-fenestrated lenses. After 3 h, the increase in fluid reservoir debris was greater for the non-fenestrated lens design (total metric increase: non-fenestrated 66 ± 21 vs fenestrated 14 ± 7 arbitrary units [*p* = 0.03], average metric increase: non-fenestrated 9 ± 2 vs fenestrated 5 ± 1 arbitrary units [*p* = 0.07]). There was no significant difference in the magnitude of debris for fenestrated lenses fitted with or without an air bubble (*p* = 0.80). There was no association between central corneal oedema and the fluid reservoir intensity metrics for either the fenestrated (*R*^2^ ≤ 0.08, *p* ≥ 0.28) or non-fenestrated lenses (*R*^2^ ≤ 0.001, *p* ≥ 0.90).

**Conclusions:**

After 3 h, non-fenestrated lenses displayed a 2 (average metric) to 4.8 times (total metric) greater increase in central fluid reservoir debris than fenestrated lenses. This suggests a potential role for fenestrated lens designs in managing fluid reservoir debris; however, longer-term studies of participants with ocular disease are required.

**Clinical Trial:**

This study was registered on the Australian New Zealand Clinical Trials Registry (Registration number 12622001164785p), date of registration 24/8/2022.

Key Points

- Limbal fenestrations may help reduce fluid reservoir debris during scleral lens wear, offering a potential strategy for managing midday fogging.
- Understanding how fluid exchange occurs beneath scleral contact lenses may guide future lens designs aimed at reducing debris accumulation and maintaining clearer vision.
- Reducing fluid reservoir debris may improve comfort and visual quality for scleral lens wearers, although longer-term studies in patients with eye disease are still required.

## Introduction

Midday fogging is an accumulation of debris within the fluid reservoir which occurs in up to ~50% of scleral lens wearers [1,2], and in some cases can result in the need for repeated lens removal, rinsing and reapplication throughout the day if vision is affected. This phenomenon is typically noted after 3–4 h of lens wear (i.e., around midday if lenses are applied in the morning); however, debris can begin to accumulate within the reservoir immediately after lens application. Fluid reservoir debris has been linked with lipids, mucins and immune cells and may be the cause [3] or consequence [4] of an inflammatory response to scleral lens wear [5].

Numerous factors are thought to contribute to midday fogging in non-fenestrated scleral lens wear, potentially including underlying ocular inflammation or disease, the lens application fluid and the thickness of the fluid reservoir [6]. Two other theories directly linking the fit of a scleral lens to fluid reservoir debris include: (1) misalignment of the landing zone with the underlying ocular surface allows the ingress of external tears, and therefore debris, into the fluid reservoir and (2) a lack of tear exchange hinders the removal of corneal metabolic waste products and debris from the fluid reservoir [6]. However, since the incidence of midday fogging appears to be independent of the extent of tear ingress in habitual scleral lens wearers [7], this perhaps favours the second hypothesis.

Fluid reservoir debris has been quantified using Scheimpflug imaging [8–10] and optical coherence tomography [11] during non-fenestrated scleral lens wear. These studies have confirmed that debris increases with wearing time, presumably since there is limited tear exchange within a sealed system to facilitate the removal of corneal metabolic waste products or mucins from the conjunctiva that migrate into the reservoir. It has been suggested that the inclusion of a fenestration may limit the extent of debris accumulation within the fluid reservoir by enhancing tear exchange [12]. However, this has not been quantified in a controlled study. Therefore, this study aimed to quantify the effect of incorporating scleral lens fenestrations on fluid reservoir debris during short-term lens wear, while controlling for other potential confounding factors (e.g., differences in landing zone alignment).

## Methods

A sample size of 20 healthy adults was selected based on previous studies which used Scheimpflug imaging to examine changes in fluid reservoir debris over time (*n* = 13–35) [8,9] and as a function of lens diameter (*n* = 15–17, 35) [8, 10].

### Scleral Lenses

Non-fenestrated scleral lenses (OneFitMed+, No.7 Contact Lens Laboratory Ltd, no7contactlenses.com) were used with the following parameters: sagittal depths ranging from 4600 to 5200 µm in 100 µm increments, 17 mm total diameter, −1.00  D back vertex power, standard prolate back surface design, 200 µm landing zone toricity (+75/−125 µm) and manufactured in Roflufocon A material. A duplicate suite of fenestrated lenses (fenestrated by the manufacturer specifically for this study) was also manufactured using the same parameters but with 3 × 1 mm diameter fenestrations positioned 6 mm from the centre of the lens at 4, 8 and 12 o’clock locations (i.e., separated by 120°).

The thickness profiles of the fenestrated and non-fenestrated lenses used in the study were determined across the central 13 mm (the approximate region covering the cornea) using an optical coherence tomographer (OCT) imaging technique [13]. This technique calculates the lens thickness along the likely path of oxygen flow through the material. The lens centre thickness requested was 260 µm, while the mean ± standard deviation measured centre thickness was 273 ± 13 µm for the fenestrated lenses and 268 ± 8 µm for the non-fenestrated lenses. The difference between the requested and measured thickness values were within the International Organization for Standardization (ISO) standard for contact lens manufacturing tolerances (±100 µm for scleral lenses) [14], and the small thickness difference (5 µm) between fenestrated and non-fenestrated lenses is unlikely to introduce a substantial difference in corneal hypoxia [15,16].

### Visit 1: Preliminary Screening

An initial ophthalmic screening examination was conducted to ensure the study inclusion/exclusion criteria were met. All participants had visual acuity of logMAR 0.00 or better in each eye, with no contraindications to contact lens wear and no history or evidence of any ocular disease, injury or surgery. Rigid contact lens wearers were excluded from participating. Six soft contact lens wearers were included but ceased lens wear for 24 h prior to each study visit.

### Visit 1: Scleral Lens Fitting

The corneal sagittal depth across a 10 mm chord along the steep corneal meridian was measured using a corneal topographer (Medmont E300, medmont.com.au) and extrapolated to a 15 mm chord by adding 2000 µm to this value. An additional 275 µm was then added to this value to determine the sagittal depth of the first scleral lens from the suite of lenses to trial on the eye (aiming for an initial central fluid reservoir thickness of ~250 µm). Fenestrated and non-fenestrated scleral lenses were then fitted to both right and left eyes with preservative-free saline (Lens Plus Ocupure, Abbott Medical Optics, abbott.com) as the application fluid. The central fluid reservoir thickness was quantified using an OCT (Spectralis, heidelbergengineering.com) and the fit of the lens was assessed using a slit lamp biomicroscope. If the initial lens selected resulted in insufficient or excessive central corneal clearance, then a different lens with a different sagittal depth was applied, and the fit reassessed. Participants then wore these lenses for approximately 30 min to ensure there was no discomfort or ingress of a central fluid reservoir air bubble. Non-central mobile bubbles that did not affect vision or comfort were considered acceptable. Half of the participants wore the fenestrated scleral lenses successfully with a mobile non-central bubble. The lens diameter, back vertex power, back surface design, landing zone toricity and lens material were held constant across all participants and between the fenestrated and non-fenestrated lens designs, and were not customised for the individual eye to account for corneoscleral shape or refraction.

### Visits 2 and 3: Fenestrated and Non-Fenestrated Lens Conditions

During Visits 2 and 3 (conducted on separate days at least 24 h apart), participants wore the optimal fitting scleral lenses (determined at Visit 1) in both eyes (e.g., Visit 2 non-fenestrated lenses in both eyes, Visit 3 fenestrated lenses in both eyes) for 3 h. To minimise the effect of any potential systematic bias introduced by the order of lens wear across the study visits, the order of lens wear (fenestrated/non-fenestrated lens) was counter-balanced across participants. Visits 2 and 3 were scheduled for the same time of day for each individual participant (at least 2 h after waking) to minimise the influence of diurnal variations in corneal thickness. Measurements of the right eye were captured using the OCT to quantify fluid reservoir thickness and corneal oedema, and Scheimpflug imaging (Pentacam HR, oculus.com) to quantify fluid reservoir debris. Scleral lenses were applied with the toric markings positioned along the 0–180 meridian and baseline images were obtained with the OCT and Pentacam. Pentacam measurements were then repeated after 30, 60, 90 and 180 min of lens wear, and OCT imaging was repeated after 180 min of lens wear for the right eye.

### Image Analysis

The OCT imaging protocol was a high-resolution volumetric scan centred on the pupil consisting of three horizontal line scans, each with 20 averaged B-scans in each line scan. Three volumetric scans were taken at each time point and exported for further analysis in customised software. OCT data were analysed by a single experienced examiner (not masked to the lens wear condition) to extract the central fluid reservoir thickness at the corneal apex and corneal thickness across the central 6 mm. Presented OCT data are the average of three repeated scans at each time point.

**Fig. 1:**
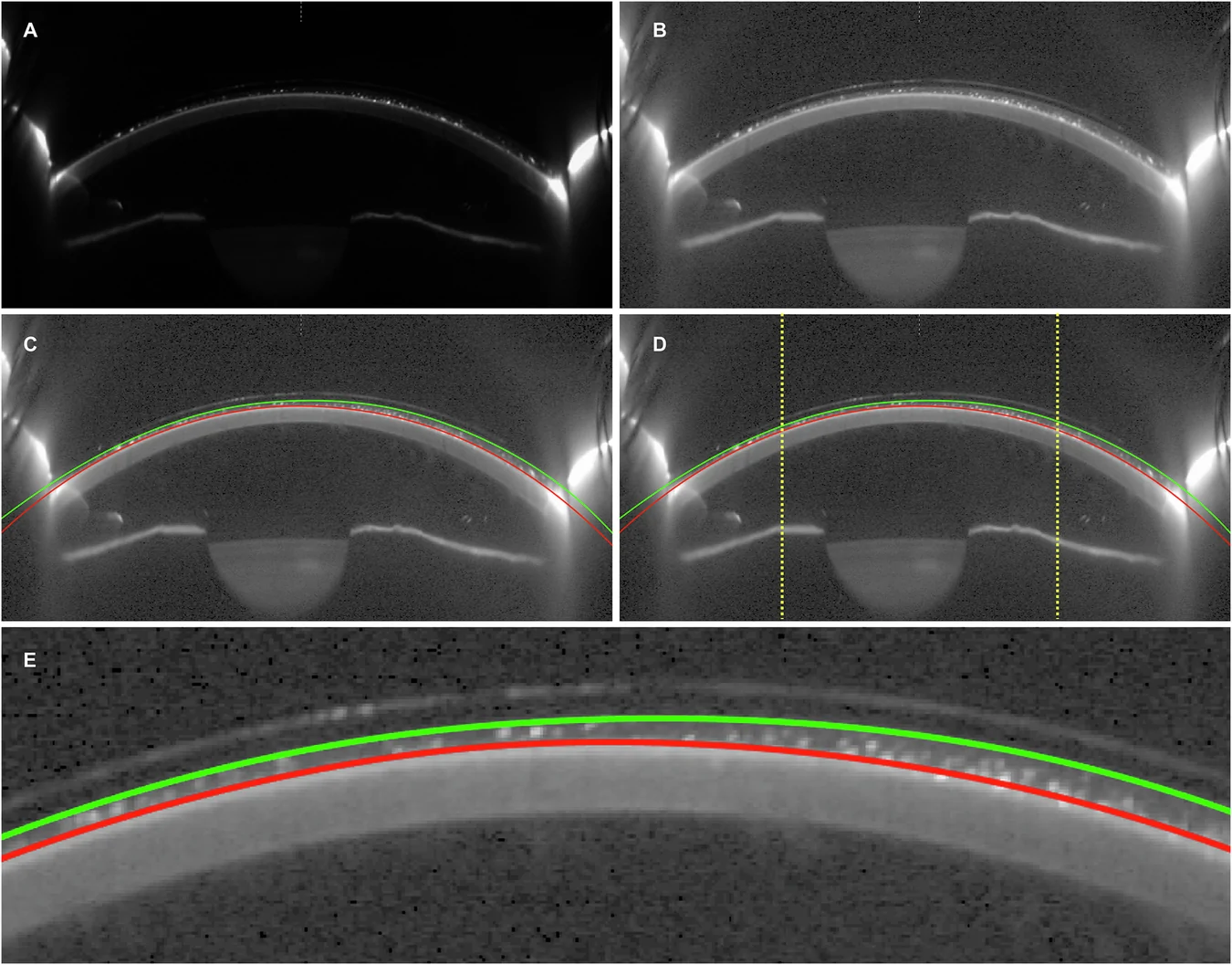
Overview of the Scheimpflug image segmentation. **A** Original Scheimpflug image exported from the Pentacam HR (oculus.com). **B** Gamma-brightened contrast enhancement. **C** Spline fits following the manual selection of the posterior scleral lens/anterior fluid reservoir interface (green line) and anterior corneal epithelium/posterior fluid reservoir interface (red line). **D** Analysis restricted to the central 6 mm of the image (yellow dotted lines). **E** Magnified image of (**D**) within the central 6 mm. The hyper-reflective pixels between the green and red lines indicate fluid reservoir debris.

To extract the debris metrics, Scheimpflug imaging was utilised (Pentacam HR 50-scan protocol). Eight radial scans (Scheimpflug images) separated by 22.5° were exported as PNG (Portable Network Graphic) files for analysis in customised software. The software allows the user to segment layers of interest within the image to demarcate the post-lens fluid reservoir for analysis. An experienced examiner (not masked to the lens wear condition) annotated two interfaces in each image: (i) the interface formed by the back surface of the contact lens and the anterior fluid reservoir and (ii) the interface formed by the anterior surface of the corneal epithelium and the posterior fluid reservoir, by manually selecting nine points along each boundary which the software then fits with a cubic spline interpolation (piecewise third-order polynomial function). The customised software has an image zoom feature, and a standard gamma contrast correction (with a gamma exponent = 3) which was used to enhance the detection of each interface (Fig. 1).

To quantify fluid reservoir debris, the intensity value was extracted directly from the original Scheimpflug 8-bit grayscale image, which has a scale ranging from 0 to 255 arbitrary units. Specifically, the intensity of each pixel along the vertical direction (per image column) between the segmented boundaries of the anterior corneal epithelium/posterior fluid reservoir interface and the back surface of the scleral lens/anterior fluid reservoir interface was automatically extracted from the image. This created intensity data for each ‘column’ of pixels along the horizontal direction. Two metrics were then calculated: (1) the average mean intensity value and (2) the sum total intensity of each ‘column’ for each horizontal pixel. To avoid light scatter from the scleral tissue and eyelid artefacts affecting the intensity of the fluid reservoir [17], the analysis was restricted to the central 6 mm.

### Statistical Analysis

Analyses were conducted with IBM SPSS Version 29 (ibm.com). The Shapiro–Wilk test indicated that the data were normally distributed; therefore, the following parametric analyses were used. For comparisons between lens conditions, a two-tailed paired *t*-test was used (e.g., for central fluid reservoir thickness comparisons between fenestrated and non-fenestrated lenses). A repeated measures analysis of variance (RM-ANOVA) was used to examine the effect of a bubble within the fluid reservoir upon fluid reservoir debris for the fenestrated lens condition only, with a within-subject factor of time (0–180 min of lens wear) and bubble (present or absent). A second RM-ANOVA (including all participants and both lens conditions) with within-subject factors of time (0–180 min of lens wear) and lens (fenestrated or non-fenestrated) and their interaction was used to examine variations in fluid reservoir debris. Bonferroni-corrected post-hoc comparisons were used for both RM-ANOVA’s. Pearson’s correlations were used to explore the association between the magnitude of central corneal oedema after 3 h of lens wear and the maximum fluid reservoir debris value across all measurements for the average and total intensity metrics.

### Ethics Approval

This study was approved by the Queensland University of Technology Human Research Ethics Committee (approval number 5469) and conducted in accordance with the tenets of the Declaration of Helsinki (Australian and New Zealand Clinical Trials Registry number ACTRN12622001164785p, date of registration 24 August, 2022). All participants provided informed consent.

## Results

Twenty young healthy adults (mean age 32 ± 11 years, 10 females/10 males) completed the study. Participants represented diverse racial and ethnic groups including Asian (*n* = 7), Caucasian (*n* = 5), Indian (*n* = 4), Middle Eastern (*n* = 2), African (*n* = 1) and Nepalese (*n* = 1).

### Central Fluid Reservoir Thickness and Corneal Oedema

The initial central fluid reservoir thickness was 258 ± 34 µm and 249 ± 39 µm for the non-fenestrated and fenestrated lenses, respectively (*t*_19_ = −0.91, *p* = 0.37), which reduced to 194 ± 33 µm and 179 ± 42 µm (*t*_19_ = −2.05, *p* = 0.05) after 3 h of lens wear (Table 1). Averaged across the central 6 mm, the mean ± SD total corneal oedema was 1.41 ± 0.84% for the non-fenestrated lenses and 0.86 ± 0.95% for the fenestrated lenses (*t*_19_ = −2.48, *p* = 0.02). Corrected central corneal oedema data, which accounts for small differences in lens and fluid reservoir thickness between the fenestrated and non-fenestrated lens conditions, have been reported previously [18] (non-fenestrated 1.32 ± 0.81%, fenestrated 0.80 ± 0.93%, *p* = 0.02), and the difference between lens designs is comparable to the raw oedema data presented in Table 1.

**Table 1:** Mean ± standard deviation central fluid reservoir thickness parameters (µm), total corneal oedema (%) and maximum debris metrics (arbitrary units) for the non-fenestrated and fenestrated lenses.

Metric	Non-fenestrated	Fenestrated	*p*-value
Initial FR thickness (µm)	258 ± 34	249 ± 39	0.37
Final FR thickness (µm)	194 ± 33	179 ± 42	0.05
Change in FR thickness (µm)	66 ± 36	69 ± 29	0.52
Total central corneal oedema (%)	**1.41** ± **0.84**	**0.86** ± **0.95**	**0.02**
Maximum FR debris (average) (AU)	19 ± 8	16 ± 5	0.09
Maximum FR debris (total) (AU)	**186** ± **98**	**135** ± **62**	**0.03**

*p*-value for the two-tailed paired *t*-test. Thickness measures at the corneal apex, oedema and debris measurements averaged across the central 6 mm. Bold values indicate a statistically significant difference between lens designs (*p* < 0.05).

*AU* arbitrary units, *FR* fluid reservoir.

### Fluid Reservoir Debris

Repeated measures ANOVA revealed that the presence of a bubble had no significant impact upon fluid reservoir debris for either the average (effect of bubble *F*_1,18_ = 0.07, *p* = 0.80, time by bubble interaction *F*_4,72_ = 1.4, *p* = 0.24) or the sum total intensity metric (effect of bubble *F*_1,18_ = 0.001, *p* = 0.98, time by bubble interaction *F*_4,72_ = 0.58, *p* = 0.68). Therefore, all participants (with and without a fluid reservoir bubble) were included in the remaining analyses.

**Fig. 2:**
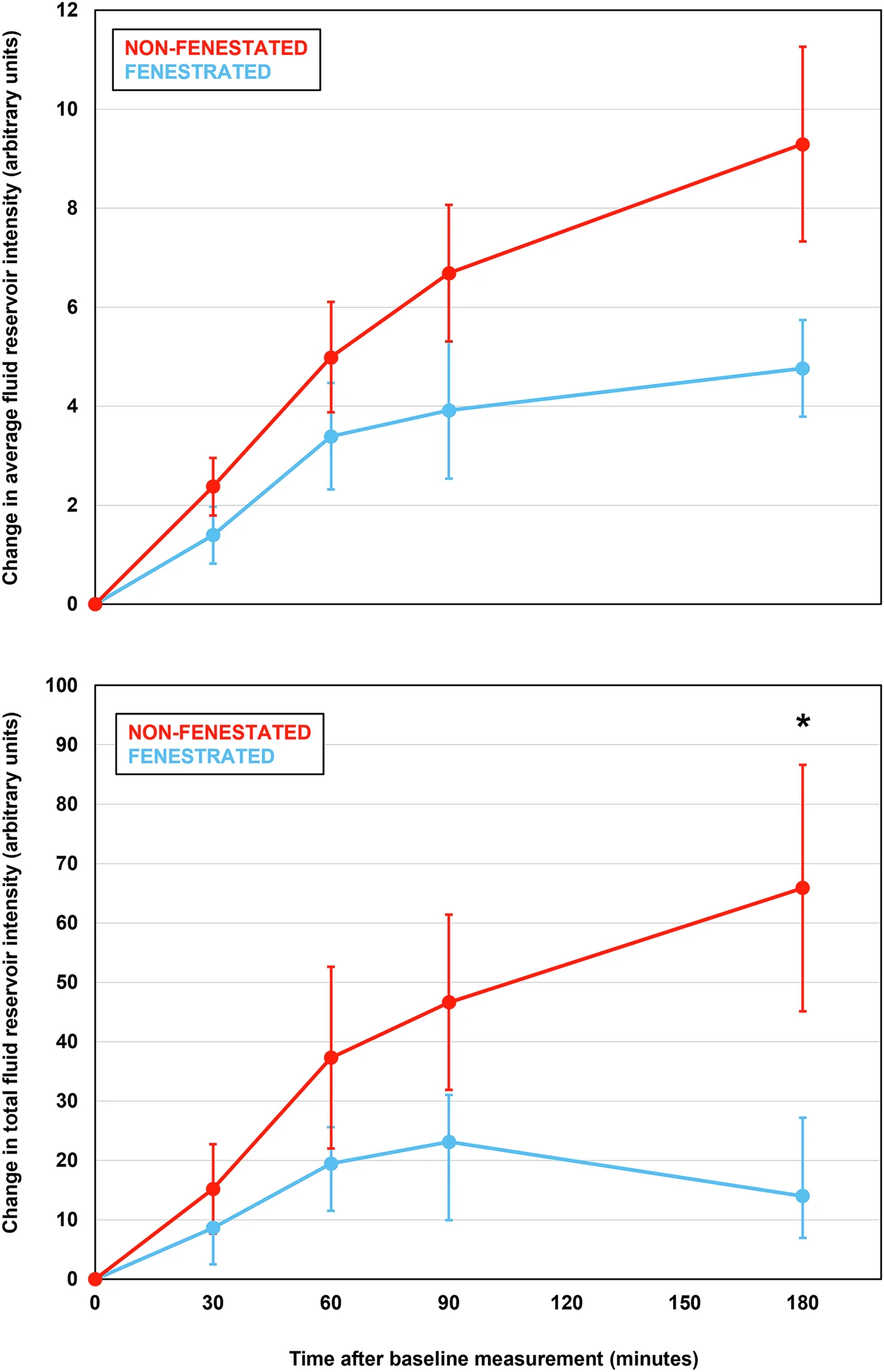
The mean change in fluid reservoir intensity (arbitrary units) relative to the baseline measurement during fenestrated (blue) and non-fenestrated (red) lens wear (*n* = 20). Error bars are the standard error. Top panel: the average intensity metric, bottom panel: the sum total intensity metric. The asterisk indicates a significant post-hoc difference between lens designs (*p* = 0.01).

The change in the fluid reservoir average and sum total intensity metrics across the central 6 mm for the fenestrated and non-fenestrated lens designs are displayed in Table 2 and Fig. 2. For the average intensity metric, there was a significant increase in fluid reservoir debris over time (*F*_4,76_ = 25.3, *p* < 0.001), but no significant effect of lens design (*F*_1,19_ = 2.32, *p* = 0.15) or a lens design by time interaction (*F*_4,76_ = 2.02, *p* = 0.10) (Fig. 2, top panel).

**Table 2:** Mean ± standard error fluid reservoir intensity metrics (arbitrary units, AU) across the central 6 mm for both the non-fenestrated and fenestrated lens designs over 3 h of wear.

	Average intensity metric (AU)			Sum total intensity metric (AU)		
Time after baseline (minutes)	Non-fenestrated	Fenestrated	*p*-value	Non-fenestrated	Fenestrated	*p*-value
0	9 ± 0	10 ± 1	0.44	99 ± 7	97 ± 6	0.73
30	12 ± 1	11 ± 1	0.60	115 ± 8	106 ± 8	0.31
60	14 ± 1	13 ± 1	0.46	137 ± 17	117 ± 10	0.20
90	16 ± 1	14 ± 1	0.22	147 ± 16	120 ± 14	0.15
180	18 ± 2	14 ± 1	0.07	**166** ± **21**	**111** ± **9**	**0.01**

The *p*-value is the Bonferroni-corrected post-hoc comparison from the repeated measures analysis of variance.

Bold values indicate a statistically significant difference between lens designs (*p* < 0.05).

For the total intensity metric, there was an increase in fluid reservoir debris over time (*F*_4,76_ = 8.14, *p* < 0.001) and more debris for the non-fenestrated lens design, averaged across all time points (*F*_1,19_ = 5.39, *p* = 0.03). A significant lens design by time interaction was also observed (*F*_4,76_ = 2.79, *p* = 0.03) (Fig. 2, lower panel), indicating that the fluid reservoir debris was significantly greater for the non-fenestrated lens after 3 h of lens wear. At the 3 h time point, the fluid reservoir debris for the non-fenestrated lens design was still increasing, but had plateaued and was decreasing for the fenestrated design. There were no significant correlations between the magnitude of central corneal oedema and the maximum fluid reservoir debris metrics (average or sum total) for either the fenestrated (*R*^2^ ≤ 0.08, *p* ≥ 0.28) or non-fenestrated lenses (*R*^2^ ≤ 0.001, *p*  0.90).

## Discussion

Although fenestrations have been utilised in contact lens practice for almost a century [19], this is the first study to examine the effect of scleral lens fenestrations on fluid reservoir debris. Three 1 mm diameter limbal fenestrations reduced the magnitude of central fluid reservoir debris (Fig. 2), irrespective of the presence of a bubble, which reached (*p* = 0.01) and approached (*p* = 0.07) statistical significance for the sum total and average intensity metrics, respectively, 3 h after lens application. In contrast to the non-fenestrated lenses, fenestrated lenses displayed an increase in debris during the first hour of lens wear which then started to plateau (average intensity metric) or decrease (total intensity metric) (Fig. 2). A repeated measures experimental design was used to limit the influence of potentially confounding variables such as landing zone design, lens application fluid and lens and fluid reservoir thickness which can impact tear ingress [20, 21] and corneal hypoxia [16, 22], and may contribute to debris accumulation [23] within the reservoir.

Consistent with previous studies [8–10], the non-fenestrated lenses displayed a steady increase in fluid reservoir debris over time, which was still increasing after 3 h of lens wear. These previous analyses [8–10], which utilised Scheimpflug imaging to quantify fluid reservoir debris, were limited to a single central point along one corneal meridian between the posterior scleral lens surface and anterior cornea using the instrument software. An advantage of the current customised approach was that the fluid reservoir was examined over a substantially larger region (the central 6 mm along eight Scheimpflug images) to provide a more robust estimate of fluid reservoir debris. Although limbus to limbus imaging of the fluid reservoir is possible with the Pentacam instrument, the analysis was restricted to the central region because scatter from the sclera and eyelids can affect the contrast towards the peripheral cornea [17], which could be incorrectly interpreted as a change in fluid reservoir intensity related to debris rather than an image artefact. Wide field anterior segment OCT imaging [24–26] may be a useful modality to examine the entire fluid reservoir without peripheral image artefacts.

Multiple factors are thought to contribute to fluid reservoir debris and midday fogging, including corneal hypoxia [6]. In the current study, 33% more central corneal oedema was observed for the non-fenestrated lens condition relative to the fenestrated condition, along with a greater maximum fluid reservoir debris for the total sum metric (Table 1). However, there were no significant associations between the magnitude of central corneal oedema and either fluid reservoir debris metric over 3 h of lens wear. This may suggest that during short-term scleral lens wear, corneal inflammation does not make a significant contribution to fluid reservoir debris, and if inflammation does play a role in generating particulate matter, then it may originate from the conjunctiva and migrate into the fluid reservoir. Conjunctival inflammation may arise during scleral lens wear due to the friction or compression from the landing zone. Additionally, patient-reported redness and irritation have been linked with midday fogging in established scleral lens wearers [2].

A limitation of the current study was the inclusion of young healthy adults only, with normal corneas and no ocular disease, combined with the relatively short duration of lens wear. Since the indications for scleral lens wear include corneal ectasia, ocular surface disease and inflammatory eye conditions [27], and some patients may wear lenses throughout most waking hours [28], the magnitude of fluid reservoir debris observed in the current study may underestimate the extent of debris accumulation in clinical populations. Future studies should examine fluid reservoir debris and visual outcomes in patients with a history of midday fogging across extended periods of lens wear to assess the effectiveness of this lens modification; potentially using a range of different fenestration sizes or configurations. Imaging techniques [29, 30] to quantify the association between fluid reservoir debris and tear exchange may also provide further insights into the management of debris accumulation and midday fogging.

In conclusion, after 3 h of scleral lens wear, non-fenestrated lenses displayed a 2 to 4.8 times greater increase in fluid reservoir debris than fenestrated lenses (based on the average and sum total intensity metrics, respectively) in healthy adults with normal corneas, when controlling for a range of other potential confounding lens and fitting factors. This suggests a potential role for fenestrated designs in managing fluid reservoir debris and midday fogging during scleral lens wear. However, longer-term studies including participants with ocular disease are still required.

## Data Availability

All data supporting the findings of this study are available within the paper.
